# Skin diseases in a primary, secondary, and tertiary setting in Trinidad and Tobago, with an evaluation of service provision

**DOI:** 10.1002/ski2.307

**Published:** 2023-11-22

**Authors:** Nirmala Hallai, Vivien Macias, Sudhir Iswaravaka, Shivaughn Ramroop, Sarah Ramjit, Priya Laloo

**Affiliations:** ^1^ Department of Medicine Eric Williams Medical Sciences Complex North Central Regional Health Authority (NCRHA) Champs Fleurs Trinidad and Tobago; ^2^ St. Joseph Enhanced Health Facility NCRHA St Joseph Trinidad and Tobago; ^3^ Chaguanas Health Facility NCRHA Chaguanas Trinidad and Tobago; ^4^ Arima Health Facility NCRHA Arima Trinidad and Tobago

## Abstract

Skin diseases in Trinidad and Tobago, demonstating differences in Hospital vs Primary Care Dermatology Clinics, paediatric age groups and use of laboratory services. This figure illustrates, (a) combined percentage in range of skin diseases from all sites, *n* = 1309. Other diseases included CTD (connective tissue disease), papular urticaria, prurigo, rosacea and pityriasis rosea. (b) Paediatric dermatology conditions seen in one PCHF (primary care health facility) versus hospital clinics. (c) Comparison of waiting times, source of referrals and use of pathology in all PCHF versus hospital clinics.

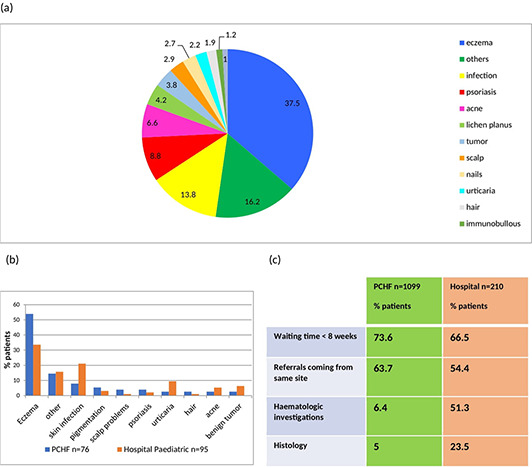

Dear Editor,

This paper aims to describe the dermatology services provided by the public health system in north‐central region of Trinidad and Tobago, commencing in 2014. The theoretical catchment population of 351 137 is an underestimation, given the availability of specialized secondary and tertiary services.

Three primary care health facilities (PCHF), two hospital clinics caring for adult and paediatric patients respectively, and an inpatient treatment unit were established. Two PCHF's had their own Accident and Emergency services, and the other provided extended hours, handling minor injuries. Utilizing available medical staff, a ‘hub and spoke’ model of care was implemented.^1^ This meant that patients not requiring secondary/tertiary hospital services, or with milder skin diseases, were managed in primary care.

Recent analysis, at the time of data collection, regarding the range of skin diseases, was limited in the Caribbean.^2^, ^3^ There is geographic ethnic diversity within the region studied, along with differences in access to Dermatology care. The collection of further information on this is essential for service evaluation and development, and this is what makes our paper unique.

A retrospective review of patients attending all Dermatology Services from October 2015 to March 2017 was done. This included new and follow up patients from the three PCHF Dermatology clinics, and two hospital clinics. Staffing included one consultant dermatologist and the equivalent of 3.5 full‐time dermatology speciality doctors.

Permission for data collection, was granted within the context of service evaluation, by the relevant health authority. Data was collected from all five clinics.

1309 patient notes were reviewed, with 63.8% female and 36.2% male. Up to 10% patients had more than one diagnoses. Eczematous skin diseases were the most common, affecting 37.5%, followed by infections in 13.8%, psoriasis 8.8%, acne 6.6% then lichen planus 4.2%. Benign and malignant primary skin tumours occurred in 3.8% of patients. Scalp, nail, and hair conditions, excluding infections, made up 2.9%, 2.7% and 1.9% respectively, Figure 1a.

**FIGURE 1:**
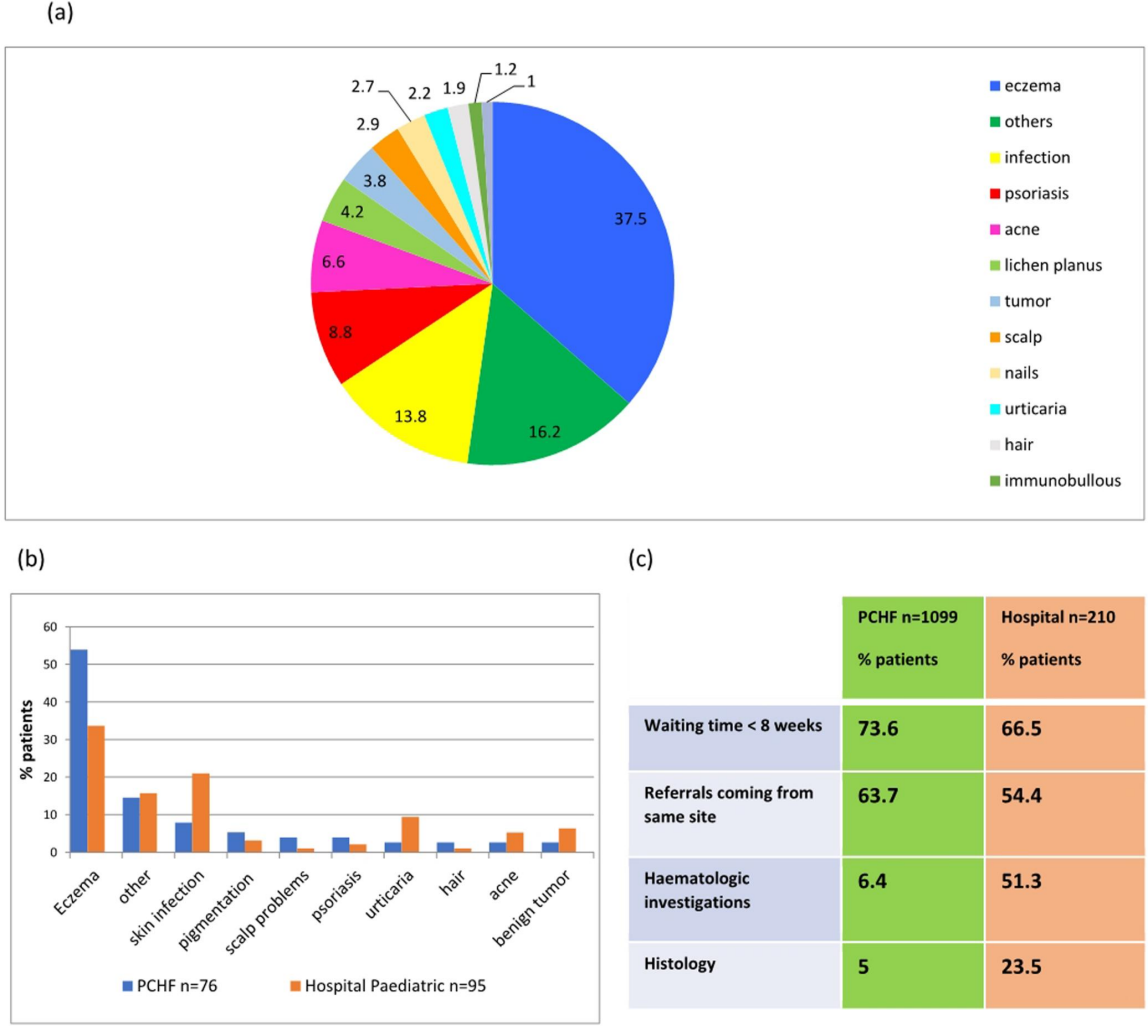
(a) Combined percent range of skin diseases from all sites, *n* = 1309 other diseases included CTD, papular urticaria, prurigo, rosacea and pityriasis rosea; (b) Paediatric dermatology conditions seen in one PCHF versus hospital clinical; (c) Comparison of waiting times, source of referrals and use of pathology in all PCHF versus hospital clinics. CTD, connective tissue disease; PCHF, primary care health facilities.

Acne was the most common condition in adolescence, and eczema was the most common in infancy and childhood, Figure 1b, in one PCHF. Fungal infections were the commonest type of infection in children attending this clinic, in comparison to bacterial infections for those attending a hospital paediatric clinic. Psoriasis was less common in children (aged <16‐year‐old), only affecting approximately 2.1%.

The adult hospital clinic managed more serious and difficult diseases. In these patients, a greater proportion had severe eczema, affecting 15%, psoriasis 14.8%, immunobullous disease 9.5% and connective tissue disease 4.3%. Other skin diseases such as vasculitis, hidradenitis, sarcoidosis and erythema multiforme etc., were diagnosed in 21.7%.

A broad classification of skin diseases was utilized to identify any trends in our population. Source of referrals, waiting times and use of pathology services, were also analyzed, Figure 1c.

The ethnic diversity of Trinidad and Tobago consists of 35.4% East Indian, 34.2% African and 22.8% mixed population.^4^ The range of skin diseases seen exhibit similar trends of inflammatory dermatoses to that observed in black people living in westernized countries.^5^, ^6^ Psoriasis was more common in areas with larger East Indian population, 13% in the central PCHF versus 6.4% and 7.4% in the other northern and eastern PCHF respectively in this region.

An earlier larger study with 7400 patients from Trinidad, reported psoriasis in 5.1% patients, due to the higher East Indian population in comparison with two studies from Jamaica, with 1000 and 547 patients respectively, with psoriasis only affecting 0.94% and 1.8% respectively. Their smaller East Indian population consisted of 3.4%.^3^, ^7^ Our population is unique in the Caribbean, having similar proportions of the three largest ethnic groups of African, East Indians and Mixed Race.

Emerging similar diagnostic trends were seen in all three PCHF's. This supports the likelihood of a true reflection of disease patterns. Less acne was seen compared to Jamaica.^3^, ^7^ This was possibly due to patients opting for local private treatment from other providers. Overall, our data is not too dissimilar to that obtained from ethnic minority living in westernized countries.^6^

Figure 1c, illustrates that hospital clinics, in comparison with PCHF's, requested more haematological investigations, 51.3% versus 6.4%, and skin biopsies, 23.5% versus 5%. Currently, the unavailability of culturing for fungi in all facilities, needs to be addressed, given its commonality. Microscopy with potassium hydroxide, can be a worthy initiative. Hospital clinics received fewer same site referrals, than the PCHF's, 54.4% versus 63.7%. This meant that they attracted more referrals from other institutions and health care providers. A greater proportion of hospital outpatients were subsequently discharged to a Dermatology PCHF than vice versa. Waiting times for first visits were satisfactory, with more than 66.5% seen in under 8 weeks.

Clinical activity has since doubled with the catchment area probably underestimated. The nationwide hospital centralization of some specialist services increases outpatient workload, attracting referrals outside the region.

The main limitations of this study were subjective variation in diagnoses, and comparatively more PCHF patients enrolled. Individuals with suspected sexually transmitted diseases, are referred to other institutions, and are therefore underrepresented here. This evaluation encourages a future, multicentre national study, using International Classification of Disease coding.

Severe skin diseases behave unpredictably and are conceptually different than primary prevention of lifestyle diseases. It is therefore imperative to develop centralized tertiary services for those requiring a specialist multidisciplinary approach. Specialty clinics in primary care elevate standards of care to the standards seen in secondary care. The main disadvantage of this structure of clinics, is the lack of not having a single designated Dermatology area. However, in a developing country, this model not only provides easier access to many outpatients, but also optimizes efficient use of limited clinic space and resources.^8^

Recognition by policy makers that Dermatology is an essential service, fosters development of subspeciality areas, training, research and staffing.

## CONFLICT OF INTEREST STATEMENT

None to declare.

## AUTHOR CONTRIBUTIONS

**Nirmala Hallai**: Conceptualization (lead); data curation (lead); formal analysis (lead); investigation (equal); methodology (lead); project administration (lead); resources (equal); software (equal); supervision (equal); validation (lead); visualization (lead); writing – original draft (lead); writing – review & editing (lead). **Vivien Macias**: Conceptualization (equal); data curation (equal); formal analysis (equal); investigation (equal); project administration (equal); software (equal); validation (equal); writing – review & editing (supporting). **Sudhir Iswaravaka**: Data curation (equal); formal analysis (equal); project administration (equal); resources (equal); validation (equal); writing – review & editing (supporting). **Shivaughn Ramroop**: Data curation (equal); formal analysis (equal); investigation (equal); project administration (equal); resources (equal); validation (equal); writing – review & editing (supporting). **Sarah Ramjit**: Data curation (equal); formal analysis (equal); investigation (equal); project administration (equal); resources (equal); validation (equal); writing – review & editing (supporting). **Priya Laloo**: Data curation (supporting); formal analysis (supporting); project administration (supporting); software (supporting); writing – review & editing (supporting).

## FUNDING INFORMATION

This article received no specific grant from any funding agency in the public, commercial, or not‐for‐profit sectors

## ETHICS STATEMENT

This data collection was approved by the Director of Health, then approved by the Chief Executive Officer, in collaboration with the Medical Ethics Committee, North Central Regional Health Authority, Trinidad and Tobago.

## Data Availability

Data available on request due to privacy/ethical restrictions.
